# Supplementary material for: Venomix: a simple bioinformatic pipeline for identifying and characterizing toxin gene candidates from transcriptomic data
Source: PeerJ. 2018 Jul 31;6:e5361. doi: 10.7717/peerj.5361 (PMC6074769; doi:10.7717/peerj.5361)
Supplement: Supplemental Information 3 [file peerj-06-5361-s003.gz › FinalOutput_E-20/Techylectin-like_protein_1/finaltree.pdf]

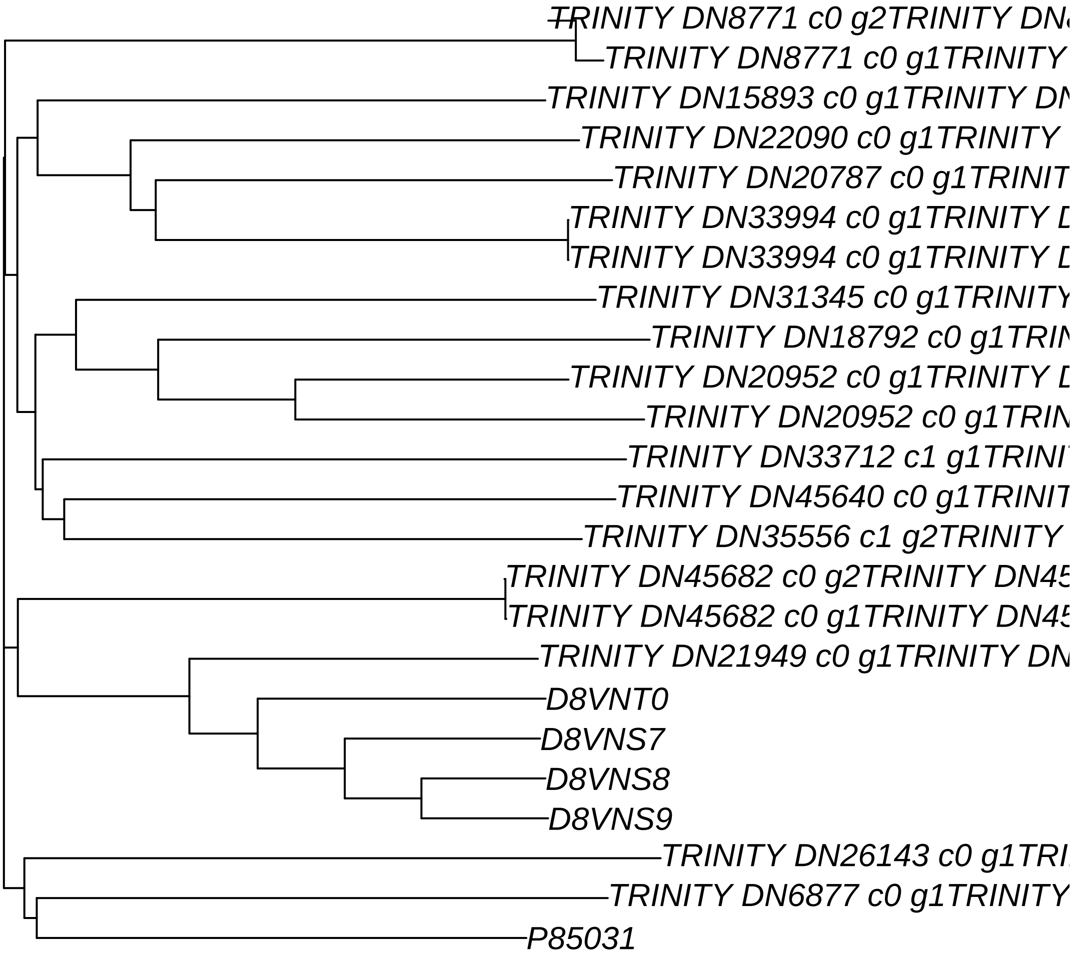
TRINITY DN8771 c0 g2TRINITY DN
TRINITY DN8771 c0 g1TRINITY
TRINITY DN15893 c0 g1TRINITY DN
TRINITY DN22090 c0 g1TRINITY
TRINITY DN20787 c0 g1TRINIT
TRINITY DN33994 c0 g1TRINITY D
TRINITY DN33994 c0 g1TRINITY D
TRINITY DN31345 c0 g1TRINITY
TRINITY DN18792 c0 g1TRIN
TRINITY DN20952 c0 g1TRINITY D
TRINITY DN20952 c0 g1TRIN
TRINITY DN33712 c1 g1TRINI
TRINITY DN45640 c0 g1TRINIT
TRINITY DN35556 c1 g2TRINITY
TRINITY DN45682 c0 g2TRINITY DN45
TRINITY DN45682 c0 g1TRINITY DN45
TRINITY DN21949 c0 g1TRINITY DN
D8VNT0
D8VNS7
D8VNS8
D8VNS9
TRINITY DN26143 c0 g1TRI
TRINITY DN6877 c0 g1TRINITY
P85031
